# Nutritional importance of animal-sourced foods in a healthy diet

**DOI:** 10.3389/fnut.2024.1424912

**Published:** 2024-07-25

**Authors:** Sydney Sheffield, Marta L. Fiorotto, Teresa A. Davis

**Affiliations:** Department of Pediatrics, USDA/Agricultural Research Service Children’s Nutrition Research Center, Baylor College of Medicine, Houston, TX, United States

**Keywords:** meat, essential amino acids, protein, diet, animal-sourced foods, nutrition

## Abstract

Animal-sourced foods, such as meats, poultry, eggs, milk, and fish are nutrient-dense foods that are rich sources of protein, essential amino acids, and micronutrients that can be challenging to obtain solely through plant-based foods. Animal-sourced protein foods provide crucial nutrients that support the growth and development in children, maintenance of muscle mass and function in adults, gain in muscle mass and strength in exercising individuals, and mitigation of sarcopenia in the elderly. The *2020–2025 Dietary Guidelines for Americans* have identified the important role of animal-sourced foods in the diet at every stage of life. Animal-sourced foods are consumed worldwide and contribute to global food security.

## Nutrient density of animal-sourced foods

Animal-sourced foods are considered nutrient-dense in that they can be a single source of high-quality protein, vitamins, and minerals; this can be challenging to obtain through consumption of only plant-based foods. Proteins are the basis of all metabolically active tissues in the body and are comprised of dispensable (nonessential) and indispensable (essential) amino acids. Dispensable amino acids can be synthesized by the body from other amino acids or nitrogen-containing molecules and do not need to be obtained from the diet whereas indispensable amino acids must be acquired through the diet. Most animal-sourced protein foods contain all the required essential amino acids in proportions that are suitable for meeting human requirements and are considered complete proteins. Many plant-derived proteins, on the other hand, contain low amounts of one or more essential amino acids such as leucine, lysine, or methionine and are considered incomplete proteins. A deficiency of a single essential amino acid will limit the use of all other amino acids for protein synthesis.

In addition to the amino acid profile of a protein-containing food, its digestibility is an important determinant of its protein quality because it determines the amount of dietary amino acid that is biologically available. Protein digestibility is determined by its amino acid composition, and protein structure can be modified by the method of processing, storage, and cooking, which also influences digestibility. Protein quality is defined by the Digestible Indispensable Amino Acid Score (DIAAS), which calculates a score for each indispensable amino acid, based on the concentration of each indispensable amino acid (per g protein) corrected for its digestibility measured at the end of the small intestine. The DIAAS for a protein is the lowest score of the individual indispensable amino acid that is most limiting as defined by its requirement in the reference population (1–3). Because most animal-sourced foods are more digestible than plant-based foods, the individual amino acids from their proteins are more readily absorbed. The combination of greater digestibility and an amino acid composition that better meets human requirements when consumed in appropriate amounts, is optimal for sustaining the body’s anabolic processes (1–3).

Animal-sourced foods are an excellent source of other nutrients that are often deficient in plant-based foods, such as zinc, iron, vitamin B12, selenium, and phosphorus (4, 5). For example, nutrient recommendations for zinc, potassium, copper, thiamin, and choline are more likely to be met in pork consumers than non-pork consumers (6). Iron, zinc, vitamin A, and vitamin D are also present in more bioavailable forms in foods from animal compared to plant sources (7). In addition, animal-sourced foods may increase absorption of iron and zinc from plant-based foods, leading to a higher probability of meeting nutrient requirements (8). However, plant-based foods do provide essential nutrients, such as dietary fiber and vitamin C that are not present in animal-sourced foods. Thus, once weaned, a healthy, balanced diet will be comprised of a variety of plant-based and animal-sourced foods that complement each other to meet nutrient requirements.

While even a food containing relatively low amounts of micronutrients or an unbalanced amino acid mixture, if eaten in sufficient amounts, will meet an individual’s requirements, the implications for total energy intake cannot be ignored. For example, for the average adult, 100 g of cooked lean beef (approximately 3 ounces) provides half of the daily needs for protein, selenium, niacin, and vitamin B12 and is an excellent source of iron and zinc, while only contributing to 10% of daily energy and fat intake (9). On the other hand, one would need to consume 250 g of peanut butter (15 tablespoons) to get the same amount of zinc, and this would provide 75% of the daily energy requirement. For adults consuming a 2,000 kcal diet, 735 g (3 cups) of low-fat milk that are recommended in the *2020–2025 Dietary Guidelines for Americans* (DGA) provides all the calcium and phosphorus, half of the protein, and a significant proportion of the riboflavin, potassium, magnesium, as well as other micronutrients required each day (4, 10). Thus, 3 servings of dairy foods help meet recommendations for nutrients which are frequently in shortfall in the diet (11). For young, physically active males, approximately 25 g of high-quality protein is needed at each meal to maximize the rate of protein synthesis in skeletal muscle (12). However, the energy content of dietary protein sources required to provide this amount of protein vary greatly. For example, 96 g (6 tablespoons) of peanut butter or 555 g (3 cups) of quinoa provide about 25 g of protein, but at a high energy value of approximately 600 kcal (4). However, only 100 g of lean beef provides the same amount of protein while contributing just 150 kcal to energy intake. Thus, lean beef has the caloric advantage of providing fewer calories but more essential nutrients, including high-quality protein.

Currently in the United States, approximately 70% of daily calories come from plant-derived foods that are derived primarily from refined grains and foods with added sugar (13). While only 30% of daily calories are obtained from animal-sourced foods, they provide nearly 100% of daily requirements for vitamin B12, calcium, and vitamin D and about 60% of requirements for zinc, iron, vitamin B6, and niacin. As Americans shift to more plant-based diets, most of the protein comes from grains, predominately refined wheat that is low in both protein quality and nutrient density, and much less from legumes that have a higher protein quality and content among the plant foods (14, 15). Thus, the reduction in nutrient density associated with a shift to a more plant-based diet will require continuous and careful consideration of diet preparation to ensure that the quantity and quality of protein consumed, and micronutrient intake meet the requirements for sustaining optimal health.

Animal-sourced foods are important for populations in low- and middle-income countries who are vulnerable to undernutrition and the consumption of these foods has been increasing (16). Using an aggregated global food composition database to calculate recommended nutrient intakes for iron, zinc, folate, vitamin A, calcium, and vitamin B12 in population groups with varying requirements, researchers found the top sources of these priority micronutrients include animal organs, beef and other meats, eggs, milk, fish, and dark green leafy vegetables (17). Populations with increased nutritional needs, such as infants and children, benefit greatly from animal-sourced foods due to the nutrient density of these foods. Indeed, addition of even small amounts of nutrient dense animal-sourced foods to the diet could alleviate deficiencies of several of “the most common shortfall nutrients in the world” (18).

## Children

Animal-sourced foods provide a rich source of nutrients that are critical at all stage of the life cycle. Nutrient-dense, protein-rich foods are critical to the growth and development of infants and children. Animal-sourced protein foods are excellent sources of many of the essential vitamins and minerals for which deficiencies are prevalent world-wide (9, 19, 20). Indeed, the World Health Organization has described animal-sourced foods as the best source of high quality nutrients to reduce stunting in toddlers and young children (21). The provision of meat as a complementary food in low-income settings is associated with less stunting in toddlers (22), and in the United States results in increased linear growth without excessive weight gain or adiposity (23). Moreover, cow milk consumption compared to plant-derived beverages is associated with greater childhood height (24). In children, consuming milk is associated with increased overall body protein balance (25), increased lean mass, and decreased body fat (26). This is particularly important when, worldwide, 20% (390 million) of children and adolescents, ages 5–19 years, are overweight and 8% (160 million) are obese. In the United States, 16% of children, ages 2–19 years, are overweight, 18% are obese, and 6% are severely obese (27, 28). Children with obesity are very likely to become obese adults and are at increased risk for cardiovascular disease, diabetes, osteoarthritis, cancer, and other chronic diseases (27). Animal-sourced foods have a role in preventing obesity in children. In overweight children, eating a high protein breakfast reduces hunger and increases fullness, fat oxidation, and energy expenditure (29), all of which are key when considering the long-term treatments for and the prevention of obesity in children. Thus, animal-sourced protein foods can play an important role in reducing the risk of obesity in current and future generations.

Malnutrition is a pervasive global health problem, especially in children. Twenty-one percent (144 million) of children less than 5 years of age are stunted, 7% (47 million) are wasted, whereas 6% (38 million) are overweight (30). Poor nutrition is associated with cognitive and behavioral impairments during adolescence and adulthood (31) and contributes to 45% of all child deaths (30). In undernourished populations, consumption of animal-sourced protein foods can promote greater length-for-age (32–34) and cognitive development and function in children (35). Thus, children in low- and middle-income countries could benefit from increased consumption of animal-sourced foods to ameliorate nutrient deficiencies and mitigate the occurrence of undernutrition (20).

## Animal-sourced foods for weight management and muscle mass anabolism in adults

In the United States, 42% of adults aged 20 years and older are obese, and 9% are severely obese (36). It is projected that by 2030, nearly 1 in 2 adults in the United States will be obese, and the prevalence will be higher than 50% in most states (37). Worldwide, obesity has doubled since 1990 and in 2022, more than 43% (2.5 billion) of adults over the age of 18 were overweight, and 16% (890 million) were obese (27). Annually, approximately 5 million deaths globally are linked to overweight and obesity (27).

The World Health Organization (WHO) recommends healthier food choices and physical exercise as the simplest techniques to prevent overweight and obesity (27). Protein rich foods can play an important role in weight management. A meta-analysis of 24 weight loss trials of approximately 12 weeks duration showed that when a portion of the carbohydrates in the diet was replaced with protein, thereby increasing the protein content from 15 to 30% of energy in the diet, participants in the studies lost more weight while preserving lean mass (38). High protein diets containing animal-sourced foods also can promote satiety, reduce food motivation and reward, and improve diet quality (39, 40).

Dietary protein is instrumental for promoting the effects of resistance exercise training on muscle protein synthesis and accretion (12, 41, 42). Consumption of egg protein after resistance exercise can promote muscle protein synthesis in a dose-dependent manner in healthy adults (12). Maximal stimulation of protein synthesis could be achieved with 25–40 g of protein, depending on protein quality (12, 43, 44). Consumption of increasing amounts of either beef alone or with exercise increases muscle protein synthesis and maximum rates of protein synthesis are achieved with 170 g of beef providing about 35 g of protein (45). Comparison of the response to 110 g (4 ounces) of beef vs. soy demonstrated that beef compared to plant-based proteins increases protein synthesis more at rest, as well as with exercise (46). Similar muscle protein synthesis rates can be achieved with 30 g of a balanced plant-derived protein blend and milk protein (47) suggesting that if plant-based proteins are combined and consumed in high doses, equivalent protein anabolism can be achieved.

The timing with which dietary protein intake is distributed over the course of the day also impacts the utilization of dietary protein for protein synthesis. Data from the National Health and Nutrition Examination Survey (NHANES) in 2017–2018 showed that most people in the United States consume protein in a skewed pattern with small portions of protein at breakfast, slightly more at lunch, and the bulk of protein at dinner (48). However, consumption of a moderate amount of high-quality protein, about 30 g, 3 times a day stimulates muscle protein synthesis to a greater extent than the common practice of skewed protein consumption (49). Thus, establishing a dietary pattern of moderate amounts of high-quality protein at each meal is a viable strategy to promote muscle mass anabolism in adults.

## Older adults

It is estimated that the percentage of adults aged 65 years and older will grow from 9% in 2015 to 17% by the year 2050 and account for 1.6 billion of the projected 9.4 billion total world population (50). Older adults may require more dietary protein than younger adults to preserve muscle mass, support health and disease recovery, offset inflammatory and catabolic conditions, protect against frailty and falls, maintain functionality, and help ensure independent living (51, 52). For example, a dose–response study investigating the effect of protein intake on muscle protein synthesis revealed that 30 g of dietary protein are needed to maximally stimulate muscle protein synthesis in older men whereas only 20 g of dietary protein are required in younger men (53). The difference in response has been attributed to the development of anabolic resistance with aging.

Age-related loss in muscle mass may be due in part to anabolic resistance to the stimulation of protein synthesis by feeding (54). However, animal-sourced foods may play an important role in promoting anabolism in muscle. Ingestion of an omnivorous meal containing beef results in higher rates of protein synthesis in skeletal muscle compared with an isonitrogenous vegan plant-only meal in older adults (55). Moreover, a higher intake of animal-sourced foods over a 20-year period is associated with the protection of muscle mass and functional performance in older adults (52). When compared to plant proteins, animal-sourced protein foods alone and with exercise are associated with the preservation of muscle mass and functional performance in older adults (56). Greater protein intake and an even mealtime distribution of protein is associated with increased muscle mass and strength in older Canadian adults (57). However, ingestion of balanced protein meals evenly distributed across the course of the day may be challenging for older adults whose energy intake is reduced. However, consumption of milk protein at breakfast or in the evening can promote higher rates of muscle protein synthesis in older adults (58, 59). Thus, supplementation with animal-sourced proteins, especially when combined with exercise, may be a viable strategy to preserve muscle mass with aging.

## Potential health risks

Red and processed meat consumption has been associated with an elevated risk of noncommunicable diseases, including cardiovascular disease, cancer, and obesity (60, 61). However, the observational data to support these assertations are weak and not confirmed by studies with more robust designs (18). Moreover, confounding dietary and lifestyle factors may also play a role in the purported link between meat consumption and disease risk. Higher red and processed meat consumption has been associated with lower vegetable and dietary fiber intakes, increased body weight, and less physical activity. Although saturated fat has been purported to be a mediator of many adverse effects of red meat on health outcomes, recent studies have called into question recommendations to limit saturated fat intake consumed within a whole food matrix such as unprocessed meat and whole-fat dairy (62, 63). A meta-analysis of 945 studies found that red meat did not influence blood lipids, lipoproteins, or blood pressure and, thus, did not adversely impact cardiovascular disease risk (64). Four systematic reviews of cohort and randomized trials of more than 6 million participants found that red and processed meat consumption has little to no effect on cardiometabolic outcomes, cancer, and all-cause mortality (65–68). Based on these systematic reviews, a panel of experts recommended that adults do not need to change their meat-eating habits (69).

Due to the above concern of the potential health risks of red and processed meat consumption, dietary guidelines have recommended the consumption of lean meat such as poultry and fish that are also a source of high-quality proteins, vitamins, and essential omega-3 fatty acids. There is some concern with fish consumption as it relates to the presence of heavy metals which accumulate in their bodies from the marine environment (70). While on average, levels are not of significant concern, for high fish consumers and pregnant women, they could pose a health risk (71).

## The 2020–2025 Dietary Guidelines for Americans

The *2020–2025 Dietary Guidelines for Americans (DGA),* published by the United States Department of Human and Health Services (HHS) and the United States Department of Agriculture (USDA), recommends that Americans “Make Every Bite Count” (10). The DGA highlights four ways to achieve this goal: following a healthy dietary pattern at every stage of life; customizing and enjoying nutrient-dense foods and beverage choices to reflect personal preferences, cultural traditions, and budgetary considerations; focusing on meeting food group needs with nutrient-dense foods and beverages, while staying within calorie limits; and limiting food and beverages high in added sugars, saturated fat, and sodium, and limiting alcoholic beverages (10). The DGA stresses the importance of nutrient density throughout the lifespan, and animal products are nutrient-dense foods that provide not only high-quality protein but also vitamins and minerals. The Guidelines recommend 735 g (3 cups) of dairy and 150 g (5 ½ ounces) of lean meats, poultry, eggs, or seafood per day in a healthy 2,000 kcal diet.

The 2020–2025 edition of the DGA is the first to investigate the nutritional habits of infants and toddlers. The Guidelines recommend feeding infants exclusively human milk during the first 6 months and supplementing with vitamin D soon after birth (10). As complementary foods are added, protein foods, including meats, poultry, eggs, seafood, nuts, seeds, and soy products, that are considerable sources of iron, zinc, protein, choline, and long-chain polyunsaturated fatty acids, should be included. In the second year of life, the DGA recommends the provision of dairy products, including milk, yogurt, cheese, and fortified soy beverages and soy yogurt, to provide a good source of calcium, along with vitamin D-fortified cow’s milk or soy beverages (10). Plant-based milk alternatives have significantly less protein than cow milk and are not natural sources of calcium. Thus, the DGA recommends cow’s milk or fortified soy beverages to meet the dairy recommendations (10).

Although the DGA uses “ounce equivalents” when recommending protein needs, animal protein and plant protein sources may not be metabolically equivalent. A recent study in young, healthy adults demonstrated that consumption of “ounce equivalents” of animal-sourced protein foods (beef sirloin, pork loin, and eggs) results in a greater gain in whole-body net protein balance than the ounce equivalents of plant-based protein food sources (tofu, kidney beans, peanut butter, and mixed nuts) and the response is correlated with the essential amino acid content of the food source (72). The improvement in whole-body net protein balance is due to an increase in protein synthesis with all the animal protein sources, whereas egg and pork consumption also suppresses protein breakdown compared with the plant protein sources.

Americans are also starting to increase their consumption of animal-sourced foods (73), similar to the global increase in consumption of animal-sourced foods (16). From 2020 to 2022, the percentage of adult respondents to a food and health survey who reported eating more red meat in the past 12 months increased from 13 to 19% (73). Additionally, about one-quarter (24%) of respondents reported that they actively tried to consume animal proteins during the studied timeframe. The percentage of consumers who perceived that animal proteins were unhealthy was only 15% in 2020. The data suggest that the majority of Americans believe that there is a role for animal-sourced foods in a healthy diet.

## Conclusion

Animal-sourced foods are nutrient-dense foods that provide high-quality protein and are rich sources of vitamins and minerals. Meat and other animal-sourced foods provide crucial nutrients that support the growth and development of children, maintain muscle mass and function in adults, and help mitigate several chronic diseases, such as those associated with aging and obesity. Thus, animal-sourced foods encompass a noteworthy role in a healthy diet.

## Author contributions

SS: Writing – original draft, Writing – review & editing. MF: Writing – review & editing. TD: Conceptualization, Writing – review & editing.
